# The joint influence of competition and mutualism on the biodiversity of mutualistic ecosystems

**DOI:** 10.1038/s41598-018-27498-8

**Published:** 2018-06-18

**Authors:** Carlos Gracia-Lázaro, Laura Hernández, Javier Borge-Holthoefer, Yamir Moreno

**Affiliations:** 1 Institute for Biocomputation and Physics of Complex Systems (BIFI), University of Zaragoza, Zaragoza, Spain; 2 Laboratoire de Physique Théorique et Modélisation, UMR8089-CNRS, Université de Cergy-Pontoise, 2 Avenue Adolphe, Chauvin, F-95302 Cergy-Pontoise, Cedex France; 3 Internet Interdisciplinary Institute (IN3), Universitat Oberta de Catalunya, Catalunya, Spain; 4 Department of Theoretical Physics, Faculty of Sciences, University of Zaragoza, Zaragoza, Spain; 5 ISI Foundation, Turin, Italy

## Abstract

In the past years, there have been many advances –but also many debates– around mutualistic communities, whose structural features appear to facilitate mutually beneficial interactions and increase biodiversity, under some given population dynamics. However, most approaches neglect the structure of inter-species competition by adopting a mean-field perspective that does not deal with competitive interactions properly. Here, we build up a multilayer network that naturally accounts for mutualism and competition and show, through a dynamical population model and numerical simulations, that there is an intricate relation between competition and mutualism. Specifically, the multilayer structure is coupled to a dynamical model in which the intra-guild competitive terms are weighted by the abundance of shared mutualistic relations. We find that mutualism does not have the same consequences on the evolution of specialist and generalist species, and that there is a non-trivial profile of biodiversity in the parameter space of competition and mutualism. Our findings emphasize how the simultaneous consideration of positive and negative interactions derived from the real networks is key to understand the delicate trade-off between topology and biodiversity in ecosystems and call for the need to incorporate more realistic interaction patterns when modeling the structural and dynamical stability of mutualistic systems.

## Introduction

Since the work of May^1^, which triggered the *complexity-stability paradox*, ecosystems research has been continuously enriched by the introduction of new paradigms aiming at understanding which mechanisms allow large and complex ecosystems to be stable^2^. While several works focus on the effect of only one type of interaction^3–6^, May’s study, which describes the ecosystem by means of a linear random matrix interaction model in which positive and negative ties are allowed, clearly stated the interest for hybrid models. Thereafter, several works have explored the joint effect of competitive or antagonistic and mutualistic interactions^7–9^. On the other hand, observations of natural mutualistic ecosystems evidenced that interaction patterns were far from being random, displaying instead a widespread signature called *nestedness*^10–13^. This particular order arises when the focus is placed on interactions, leading to a new paradigm under the light of network theory. Most often these networks are bipartite, as in the frequently studied cases of plant-pollinator (or plant-seed dispersers) systems. The two disjoint sets of vertices of such network, correspond to plant species and pollinator (or seed-dispersers) species, and the links which stand for the mutualistic interactions, only connect vertices of different kind^14–17^.

**Figure 1:**
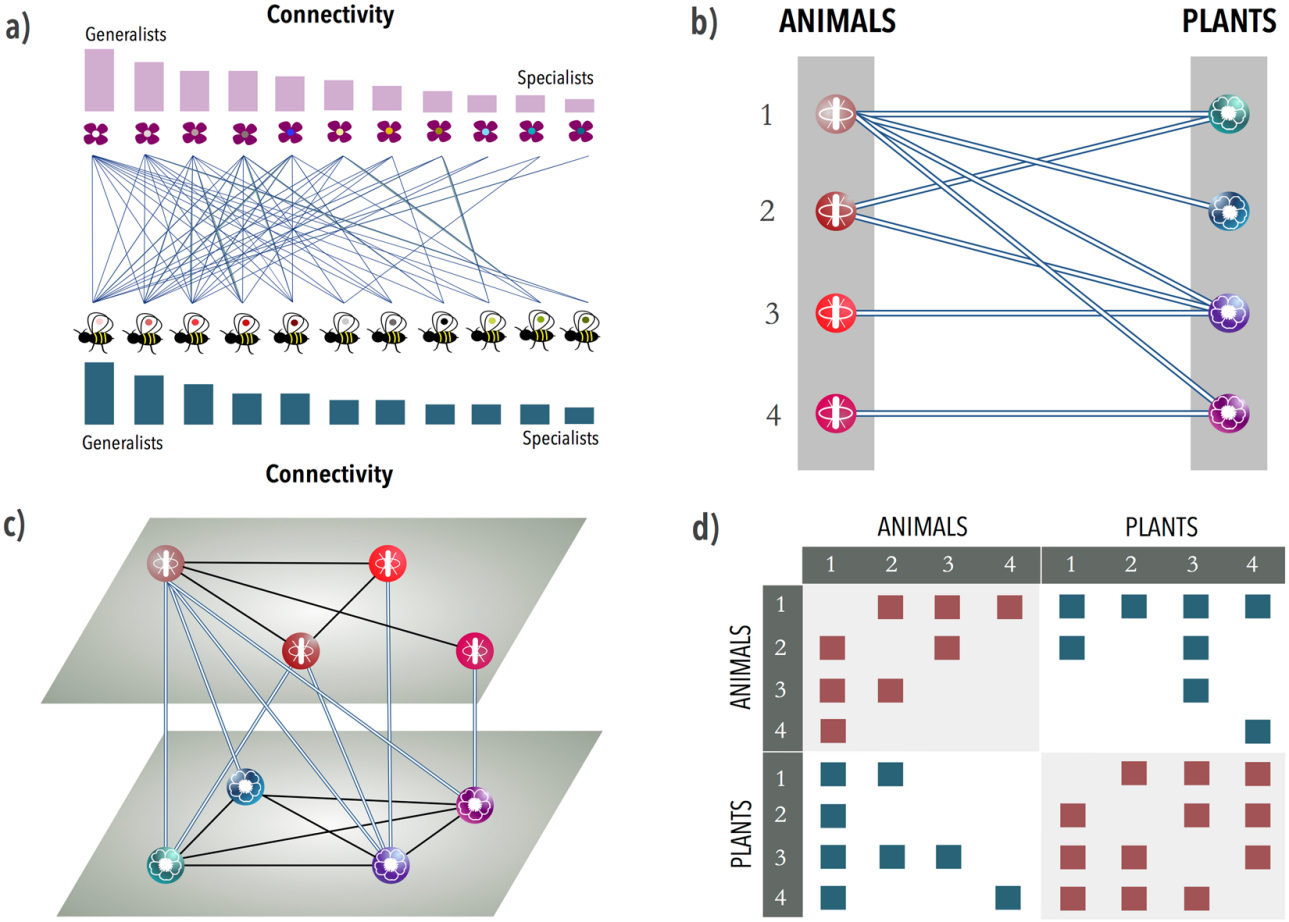
Multilayer mutualistic network. Panel (a) illustrates a mutualistic system made up by plant and animal species. In this representation, mutualistic interactions are given by the inter-connections among the elements of a bipartite graph, as shown in panel (b) for a synthetic network. Generalists have a higher connectivity than specialists. This representation does not account for intra-guild interactions. Panel (c) illustrates the multilayer approach proposed here for the ecosystem of plants and animals of panel (b), which consists of 4 species of each guild. In this framework, each layer represents one guild and an intra-layer link exists whenever two species of the same guild share the same species of the other guild. These links represent the competition among species of the same guild that are mediated by the mutualistic connections. These two layers are coupled by the mutualistic interactions given by the bipartite graph. Finally, in panel (d) we depict the adjacency matrix of the whole system, including both plant-plant and animal-animal competitive interactions (elements of the diagonal blocks in red) in addition to the usual mutualistic links (elements of the off-diagonal blocks in blue).

Such network is said to be nested when the contacts of a species of a given degree are a subset of the contacts of all the species of higher degree. Thus a nested system is composed of specialist and generalist species of two guilds (having a small and a large number of inter-guild interactions, respectively, see Fig. 1a,b), with specialist-specialist interactions being rare. The ubiquitous character of nestedness in mutualistic ecosystems prompted a new debate about its origin and its role in the preservation of biodiversity, which is still open^18–21^. In particular, it led to a reconsideration of May’s ideas towards structure-sensitive dynamics. As a result, it is now generally accepted that structure (nestedness) and dynamics (as given by the persistence or biodiversity of species) of mutualistic ecosystems are intimately connected^17^.

While the bipartite network representation of the system is ahead of any random structure assumption, it still deals with only one type of interaction, mutualism, leaving aside intra-guild competitive links. To overcome this problem, intra-guild competition was introduced via a *mean field approximation*^17^ (MF). In this framework, each species of a given guild is assumed to interact equally with all the others in the same guild. Thus, far from being a parsimonious hypothesis, the MF approach actually involves a very strong one: every species interacts with all the others, and with the same strength, thus ignoring competitive interactions that are mediated by species of the other guild (i.e., plant-plant interactions mediated by a shared pollinator and vice versa). It is well known in Physics that considering a fully connected network is equivalent to the statistical treatment of one single element under the effect of a field that accounts for the average interaction created by all the others, leading to a description that underestimates the entropy of the system^22^.

In order to explore the relationship between structure and dynamics, it is then imperative to properly deal with both positive (mutualistic) and negative (competitive) interactions in a way that naturally allows to plug dynamical population models in. To this end, the recently developed framework of multilayer networks^23^ provides a straightforward representation of the system, allowing to encode *both* kinds of interaction within a unique topological representation. In fact, this framework has been successfully applied in problems as diverse as percolation of interdependent systems^24^, diffusion processes^25^ and disease spreading^26,27^ as well as to study transportation systems^28^ and evolutionary game dynamics^29^. In particular, mutualistic ecosystems fit naturally in a structure consisting of two layers, each one containing nodes of a different guild, with intra-layer links representing competitive interactions between species of the same guild, and the nodes of different layers being coupled by inter-guild mutualistic interactions –what technically corresponds to a *network of networks*^30^–, see Fig. 1c. This allows us to study, analytically and numerically, how the biodiversity of the system varies as a function of the intensities of mutualism and competition in the system.

## The model

In order to build model that treats the intra-guild competition beyond the mean field approximation, it is essential to focus on the possible sources of heterogeneity affecting such competition. Species of the same guild compete for abiotic resources, like water, soil nutrients, light, etc. This competition may well be approximated by a mean field term affecting all the species of a each guild, on average, in the same manner. However, when it comes to competition for the services furnished by counterparts (either pollination services or food), it is easy to understand that heterogeneities may become important. For example it is clear that two plant species pollinated by two disjoint sets of pollinators do not compete for pollination services at all. Moreover, it is important to notice that the competition between two given species is not symmetric. Let us consider two plant species that share the same pollinator, one being a generalist and the other a specialist. It is clear that the latter will be more affected than the former, because it needs to share its only resource while the generalist may compensate by the services furnished by its other counterparts.

Interestingly, the necessary information is partially encoded in the bipartite matrix: the projections onto the animals and plant subspaces yield two hidden weighted networks that reveal the existent intra-guild interactions, see Fig. 1c. In other words, two plant (animal) species will be connected (competing) in the corresponding plant (animal) layer if they share at least one pollinator (plant). In this way we are able to model inter-species competition beyond the mean-field approach, i.e., considering the actual architecture of intra-layer interactions, and thus integrating the necessary (though not sufficient) condition of niche overlap. This is a more realistic scenario for competition (with respect to mean field), i.e., it restricts the competitive term to those species with non-zero niche overlap. As such, it mitigates competition overestimation; but it does not eliminate it completely, e.g. species of the same guild, with niche overlap which do not overlap in time would actually have an even lower competition. Finally, the two layers are coupled by the mutualistic interactions, see Fig. 1c,d. All this information is coded in the multilayer adjacency matrix (Fig. 1d). In this way, the multilayer representation takes into account the two aspects of the interaction in mutualistic ecosystems, the benefit obtained by species of different guilds, along with the intra-guild competition that arises when two species share the same counterparts.

We next investigate the influence of the network structure on the persistence of species of the mutualistic ecosystem. The main point of interest is whether the competitive interactions, as given by the multilayer topology, actually convey substantive dynamical changes. To this end, and in order to compare with previous results, we implement a population dynamical model that builds on the one introduced by Bastolla *et al*.^17^, where we explicitly introduce the multilayer architecture. We study the variation of biodiversity (e.g., the number of species at the steady state of the population dynamics) on a set of large real mutualistic communities^31–43^ (see Tables S1–S2 and Fig. S4 in the Supplementary Information).

Let’s assume that the mutualistic community consists of ${N}^{P}$ species of plants and ${N}^{A}$ species of animals (pollinators or seed-dispersers); the biodiversity is denoted by $N={N}^{P}+{N}^{A}$. We denote by ${s}_{i}^{P}$ the abundance of the plant species $i$, and by ${\alpha }_{i}^{P}$ its intrinsic growth rate. Similarly, animals’ parameters and variables are represented by the superscript $A$. The mutualistic relationships (inter-layer connections) are given by a rectangular ${N}^{P}\times {N}^{A}$ matrix, $K$, with ${K}_{ik}=1$ if animal species $k$ pollinates the plant species $i$, and ${K}_{ik}=0$ otherwise. The total abundance of the pollinators of a given plant species $i$ is thus ${M}_{i}^{P}={\sum }_{k\in A}{K}_{ik}{s}_{k}^{A}$. On the other hand, the intra-layer relationships represent the resources that are shared by species of the same guild. Therefore, the abundance of the pollinators shared by two plant species $i,j$ is ${W}_{ij}^{P}={\sum }_{k\in A}{K}_{ik}{K}_{jk}{s}_{k}^{A}$. Finally, the relative abundance of a given plant $i$ evolves according to:

1 $$\frac{1}{{s}_{i}^{P}}\frac{d{s}_{i}^{P}}{dt}={\alpha }_{i}^{P}-{\beta }_{i}^{P}{s}_{i}^{P}-{\beta }_{0}^{P}\frac{\sum _{j\in P,i\ne j}{s}_{j}^{P}{W}_{ij}^{P}}{{M}_{i}^{P}}+{\gamma }_{0}^{P}\frac{{M}_{i}^{P}}{1+{h}^{P}{\gamma }_{0}^{P}{M}_{i}^{P}}$$

The first term of this equation represents the intrinsic growth of the abundance of species $i$ without considering saturation and the second term refers to the intra-specific competition term (saturation), which can be interpreted in terms of a carrying capacity in the absence of competing species. The third term of equation 1 accounts for the intra-guild, inter-specific competition (represented by the intra-layer links). Here, the competition between two plant species ($i,j$) is weighted according to the relative importance of shared pollinators with respect to the total abundance of pollinators of plant species $i$. Lastly, the fourth term in equation 1 gives the contribution of mutualism to the abundance of plant species $i$, ${h}^{P}$ being the Holling term that imposes a limit to the mutualistic effect. The intensities of competition and mutualism ${\beta }_{0}$ and ${\gamma }_{0}$ respectively, constitute the parameter space that we investigate. The corresponding equation for the abundance of pollinators is equivalent to equation 1 but interchanging superscripts $P$ by $A$ and vice versa (see equation 3 in Methods).

## Results

**Figure 2:**
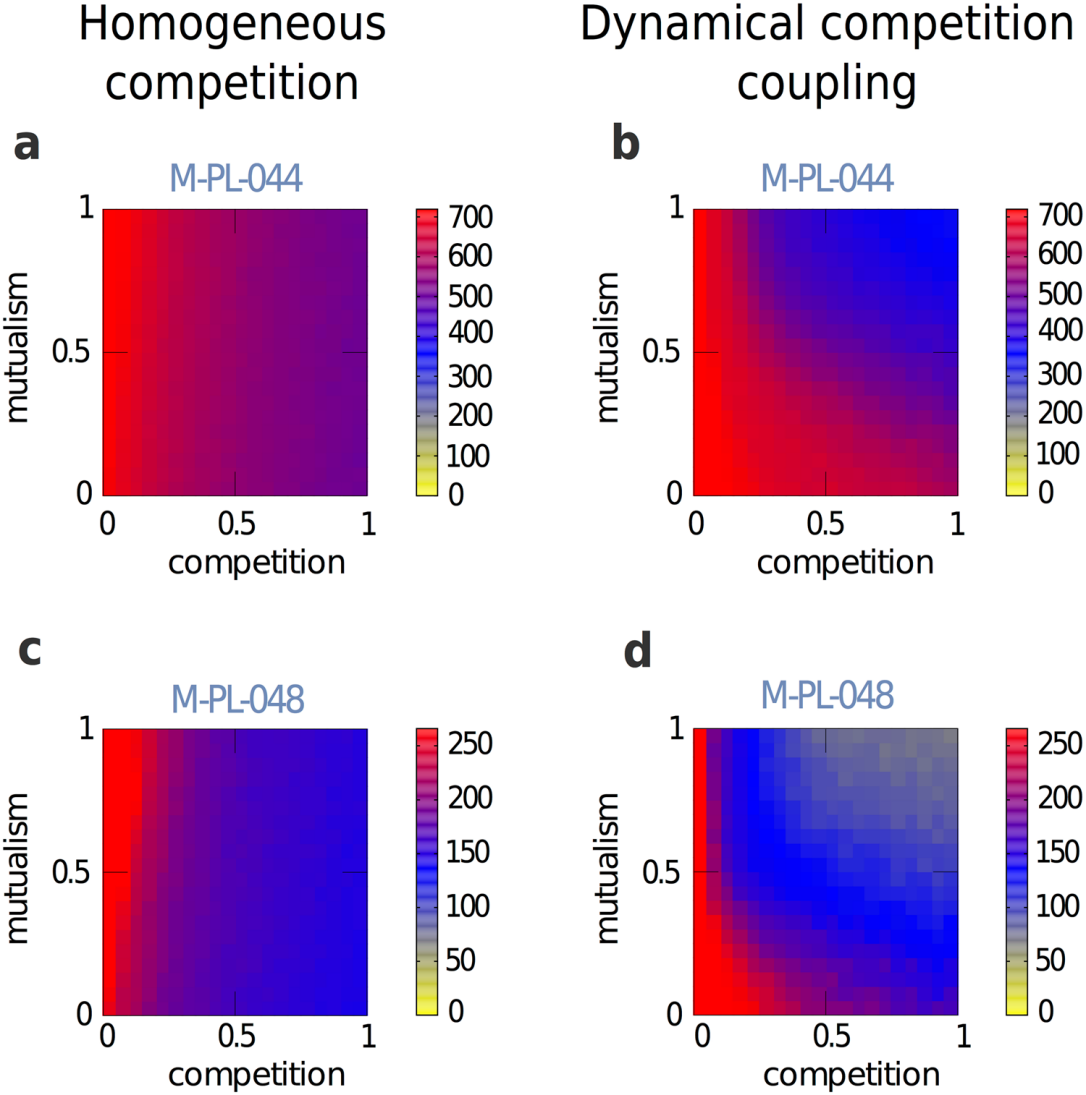
Biodiversity as a function of the mutualism and intra-guild competition parameters. We show the results obtained by numerically simulating the dynamical population model where the network’s structure corresponds to two real mutualistic systems. The levels of biodiversity are shown as a function of the intensities of mutualism and competition (the maxima being $N=719$ for M-PL-044 and $N=266$ for M-PL-048). The color scale represents biodiversity, given by the number, $N$, of species present in the steady state. Left panels a and c show the results obtained when the system evolves according to a dynamics that corresponds to the simplified model given by eq. 2. Right panels b and d show the results obtained for the model corresponding to eq. 1. We show results for two different real networks: M-PL-044^37^ in top panels a and b; and M-PL-048^39^ in bottom panels c and d. The characteristics of these networks as well as more results for other networks are presented in the Supplementary Information (Tables S1, S2 and Figs S1 to S3). These results correspond to the following parameter values: intra-specific competition terms ${\beta }_{i}^{P}={\beta }_{k}^{A}=5.0$, growing terms ${\alpha }_{i}^{P}\in \mathrm{(0.9,}\,\mathrm{1.1)}$, ${\alpha }_{k}^{A}\in \mathrm{(0.9,}\,\mathrm{1.1)}$ and Holling terms ${h}^{P}={h}^{A}=0.1$.

We numerically solve the system of equations describing the abundances of plants and animals (see Methods). Figure 2 compares results obtained for the system’s biodiversity as a function of the intensities of mutualism and competition. Left panels (a,c) correspond to the homogeneous case where the existing competition terms have the same weight, regardless of the abundances of the shared counterparts. In other words, we have considered *binary* projected matrices, which correspond to the situation where two species of the same guild compete with the same intensity whenever they share *at least* one counterpart. This simplifies the second term of eq. 1, which becomes:

2 $${C}_{ij}=-\,{\beta }_{0}^{P}\sum _{j\in P}{s}_{j}^{P}{\tilde{V}}_{ij}$$

where ${\tilde{V}}_{ij}=1$ if plant (animal) species $i$ and plant (animal) species $j$ share at least one pollinator (plant) and zero otherwise. On the right panels the results shown correspond to the model given by eq. 1, where the topological structured competition is weighted by the abundances of the shared counterparts. These models are implemented on two real networks: M-PL-044^37^, with $N=719$ species, in panels a and b; and M-PL-048^39^, with $N=266$ species, in panels c and d. It clearly appears that when only the topology of the intra-guild layer is introduced in the competition term, the persistence of biodiversity in the real systems does not depend on ${\gamma }_{0}$. Conversely, when the abundances of shared counterparts are taken into account, the region of structural stability depends, non-trivially, on both parameters, ${\gamma }_{0}$ and ${\beta }_{0}$, (see also Figs S1–S3 in the Supplementary Information for results corresponding to other real networks).

The results shown in Fig. 2 may appear counterintuitive. Indeed, if mutualistic interactions were to reduce effective competition and increase biodiversity^17^, one should expect that the boundary separating the region where all species survive (coded in red in Fig. 2) from the one where biodiversity diminishes (coded in blue in Fig. 2) would behave as a monotonous growing curve, which is clearly not the case.

**Figure 3:**
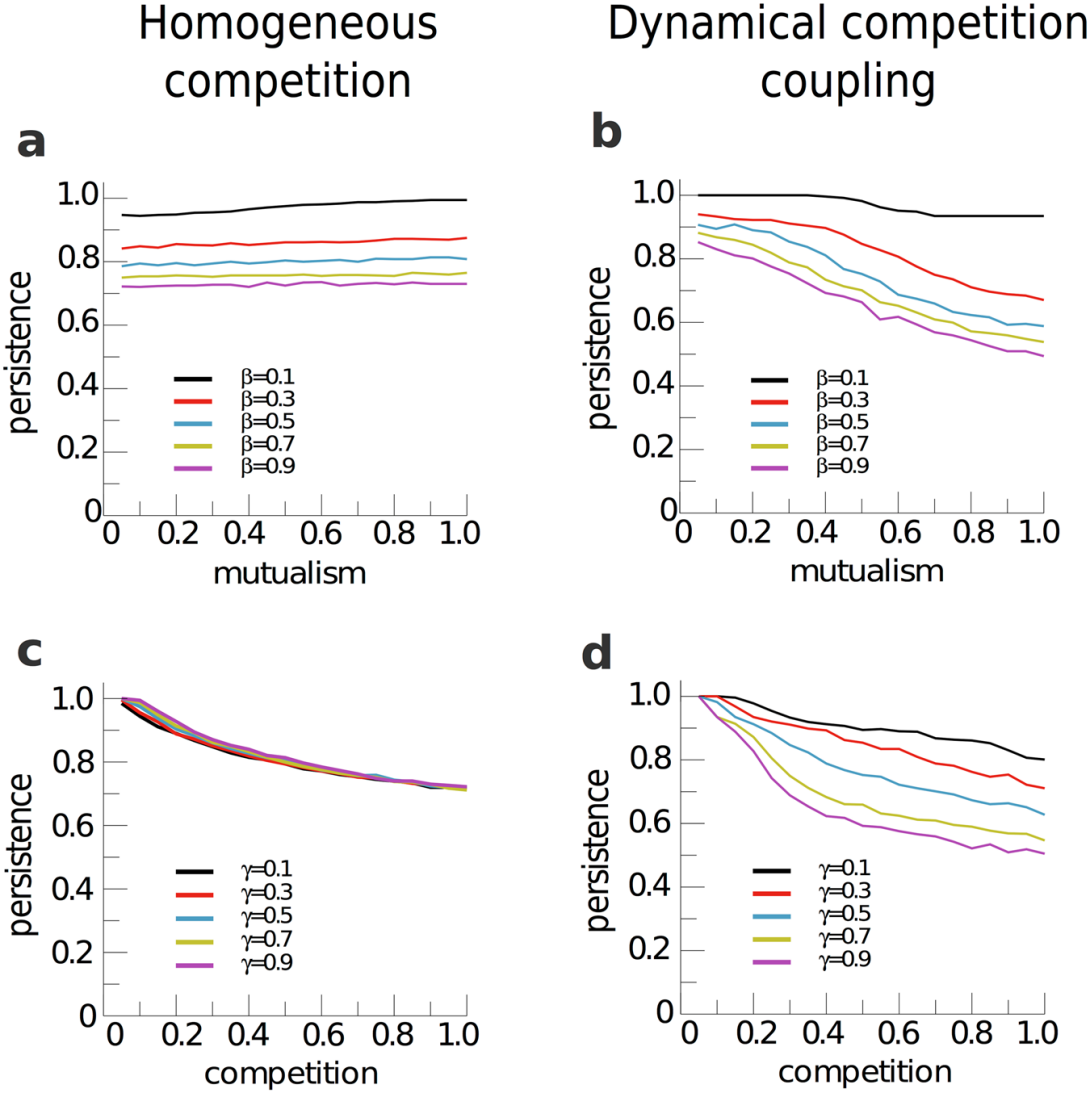
Persistence of biodiversity. Top panels a and b show the fraction of species in the steady state as a function of the intensity of mutualism for different constant values of the competition intensity ${\beta }_{0}$. Complementary, bottom panels (c and d), show the persistence of biodiversity as a function of the competition intensity, for different values of fixed mutualism, ${\gamma }_{0}$. Left panels a and c correspond to the bipartite representation of the mutualistic systems, in which the competitive interactions are given by the simplified model with homogeneous competition intensity; whereas right panels b and d show results obtained when the dynamical population model is constrained by a multilayer network, thus properly accounting for the competitive interactions weights. These results correspond to the network M-PL-044.

Figure 3 further illustrates this point. As expected, the biodiversity is a decreasing function of the competition parameter (${\beta }_{0}$) in both settings when the mutualism intensity (${\gamma }_{0}$) is fixed (panels c and d). Note, additionally, that in the case in which competition intensity is homogeneous, the persistence is independent of ${\gamma }_{0}$, as also shown in Fig. 2. Remarkably enough, when the relative weights are accounted for in the competition term, the same decreasing behavior for the persistence of species is observed when the intensity of competition is kept constant and the mutualistic parameter is increased, see panel b in Fig. 3, except for very low ${\beta }_{0}$ values, where competition may be neglected and biodiversity hardly varies with mutualism.

**Figure 4:**
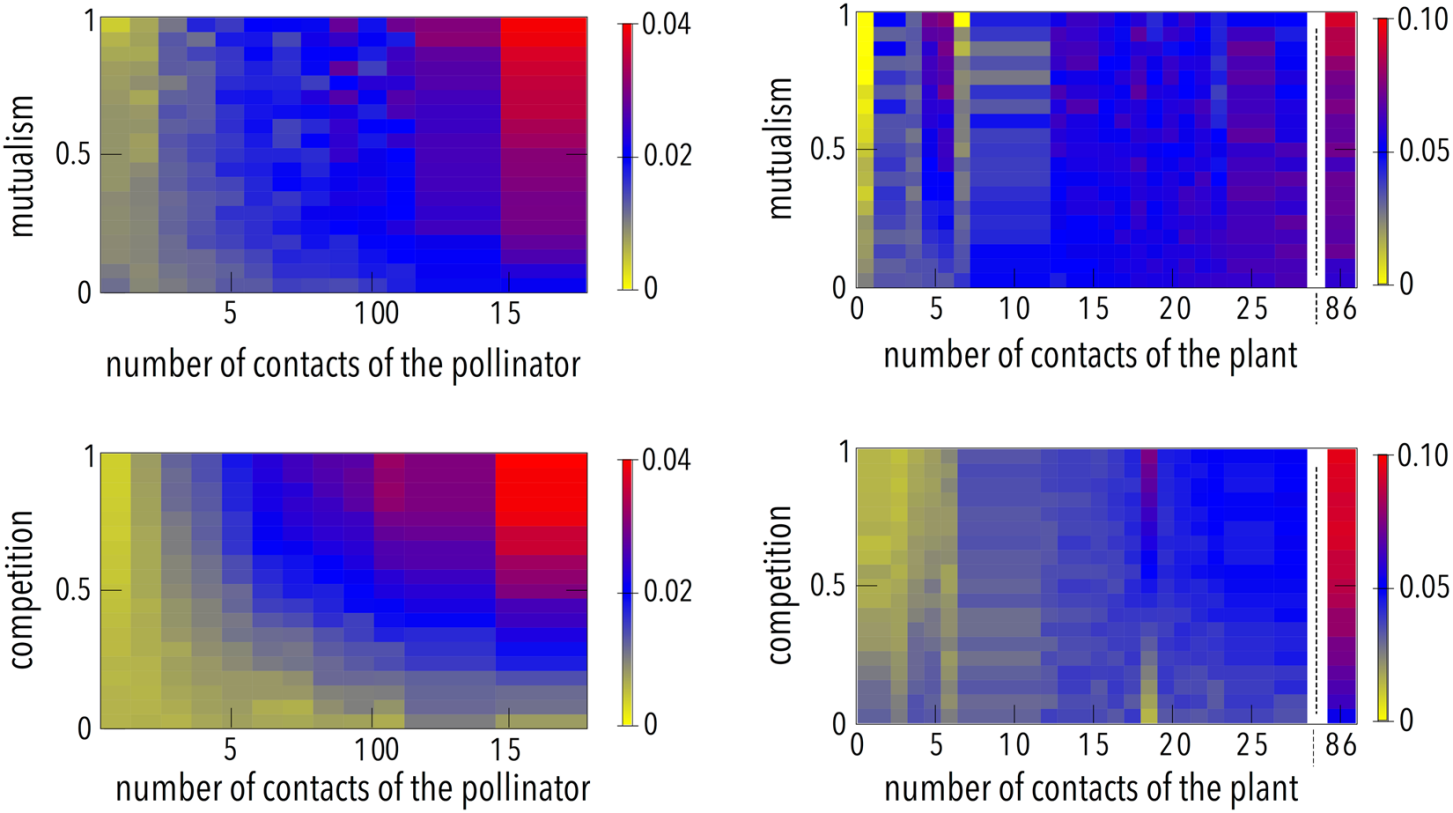
Relative abundance of the species according to their connectivity, in the multilayer model. The results shown here correspond to the evolution of the system according to equations 1 and 3, with the interaction terms constrained by the real network M-PL-016 (left panels: pollinator species; right panels: plant species). Top panels: The competition intensity parameter is fixed to ${\beta }_{0}=0.25$, the color scale represents the relative abundance of the different species, as a function of the mutualism parameter, ${\gamma }_{0}$, and inter-layer connectivity. Bot- tom panels: The mutualism interaction is fixed to ${\gamma }_{0}=0.25$, the color scale represents the relative abundance of the different species, as a function of the competition parameter ${\beta }_{0}$ and interlayer connectivity. The gaps on right panels correspond to non existing values of the plant’s degree. Animals (left panels) and plants (right panels) have been ranked in ascending order of interlayer degree. The rest of parameters are the same as in Fig. 1.

This paradoxical result is due to the joint action of the mutualistic and the inter-species competition terms, which lead to differences in the way the abundance of generalist and specialist species evolve (see Methods for an heuristic argument and section 5 of Supplementary Information). The selective influence of both mutualism and competition on specialist and generalist species is illustrated in Fig. 4, where we represent the relative abundance of the species as a function of the species’ connectivity and the intensities of mutualism (top panels, in which competition is fixed) and competition (bottom panels, in which mutualism is fixed). The results are clear-cut: it turns out that species with higher degrees remain relatively more abundant than those with lower degrees when there is an increase of the strength of either mutualistic or competitive interactions. A similar paradoxical result, where an increase of mutualism leads to a decrease of biodiversity has been observed in the system composed by plants and mycorrhizal fungi^44,45^.

The results that come out from the approach adopted here unveil the important role played by the network structure of the inter-species competition term on the biodiversity of the system. When this interaction is treated homogeneously, biodiversity persists for any intensity of mutualism, as long as the inter-specific competition remains under a certain value –the frontiers indicating the loss of biodiversity are vertical in panels a and c of Fig. 2. On the contrary, when considering the network structure in the mutualistic *and* in the intra-guild competition term properly weighted by the shared resources of the competitors, the region of the parameters space where the biodiversity persists depends non-trivially on the intensity of both. In other words, increasing the intensity of mutualism (for a given network) does not necessarily increase biodiversity. Indeed, depending on the intensity of competitive interactions, higher levels of mutualism are detrimental for the survival of the (specialist) species.

The results displayed in Figs 2–4 hold in different scenarios, which are developed in the Supplementary Information. It is proved there (section 2) that results do not stem as a mere consequence of the heterogeneity in the distribution of inter-species competition constant; they are a consequence of weighting the interaction by the abundance of the shared resources, which reinforces the coupling of the equations. The SI (section 3) also shows that results are robust against the size and the density of contacts of the mutualistic networks, and against a wide range of values of the Holling constant, that controls the saturation of the mutualistic term (see Fig. S5 in SI). Moreover, we have verified (section 4 of the SI and Fig. S6) that the inclusion of an extra inter-species competition term, representing abiotic competition sources (e.g. water or light), does not alter the observed behavior. Hence, we conclude that this behavior is a consequence of the structure of the interactions. Noteworthy, our findings challenge the idea that in mutualistic ecosystems, which present nested architectures, the mutualistic interactions help to screen competition, thus enhancing biodiversity^12,14,17,46,47^. Within our model, this statement is only roughly valid for weak competition levels, when increasing mutualism is not detrimental to biodiversity (see Fig. 3).

However, our results do not imply that mutualism is not relevant in order to explain the existence of large complex ecosystems. Instead, the careful treatment of the structure of the interactions provides a better understanding of the subtle trade-off between competition and mutualism. In ref.^47^ an indirect mechanism for cooperation via the interaction with a common counterpart was discussed. Here we show, in addition, that the asymmetry of the competition term between a generalist and a specialist, induced by mutualism, favors the generalist species. This explains why when the intensity of the mutualistic interactions increases, biodiversity may diminish through an important loss of specialists species in favor of the increase of the population of the generalist ones. Moreover, we have checked that our conclusions are indeed due to the structure of the inter-species competition term and not to the fact that the intensity of this competition is just inhomogeneous. That is, one might think that similar results could be obtained using heterogeneous intensities in a competition term of the form given by eq. 2. The results (details can be found in section 2 of Supplementary Information) indicate that this heterogeneity is not enough to reproduce the same patterns of biodiversity obtained when the model given by eq. 1 is considered. Similar independent findings have been reported –though limited to the particular case of weak competition^48^. In that work, as in the previously described test, the structure of the competition term is not taken into account; instead each species compete with all the others within the same guild but with a random intensity of the interactions. Finally, we point out that the next interesting step would be to filter out competitive interactions that we might still be overestimating. To this end, it is key to obtain and include more data regarding actual niche overlap in space and time in real ecological networks. Ours is a first step towards this goal, and lay the groundwork for more refinements.

## Conclusions

In summary, we have introduced a network representation for mutualistic systems that further exploits the information encoded in the bipartite mutualistic graph. We have shown that this multilayer network better displays the structure of both mutualistic and competitive interactions among species within a unique representation. Numerical simulations of a dynamical population model that is coupled to the data-driven multilayer architecture revealed that biodiversity persistance is not just a consequence of the screening of competition by mutualism, as previously reported^17,46^. Instead, our results show that the network topology induces a complex trade-off between mutualism and competition, which differently affect species according to their degree. Strikingly, we have shown that, contrary to what one would have expected, when the level of competition is high, the biodiversity of the system is higher for lower mutualistic intensities and indeed, increasing mutualism maybe detrimental for the species persistence. In light of the present results, there are a number of further questions that remain to be explored, including whether there is an optimal value of mutualism (and competition) at which the system maximizes biodiversity and its dependency with the nestedness of the system. Considering the approach introduced here would helpfully provide more realistic grounds to tackle these and related challenges.

## Methods

### Equation for species A

The dynamical equation that describes the evolution of the abundance of animal species $i$ is equivalent to equation 1 in the main text, i.e.,

3 $$\frac{1}{{s}_{k}^{A}}\frac{d{s}_{k}^{A}}{dt}={\alpha }_{k}^{A}-{\beta }_{k}^{A}{s}_{k}^{A}-{\beta }_{0}^{A}\frac{\sum _{l\in A,k\ne l}{s}_{l}^{A}{W}_{kl}^{A}}{{M}_{k}^{A}}+{\gamma }_{0}^{A}\frac{{M}_{k}^{A}}{1+{h}^{A}{\gamma }_{0}^{A}{M}_{k}^{A}}$$

### Numerical simulations of the model

We numerically solve the system of equations 1 and 3, using the matrices ${K}_{ik}$ that correspond to 14 different real systems^31–43^ (see Tables S1 and S2 in the Supplementary Information). Each simulation starts from random initial conditions of the relative abundances that are taken at random from a uniform distribution between 0.05 and 0.95. Following refs^17,46^ we take the values of ${\alpha }_{i}^{P,A}$ from a uniform distribution in the interval [0.9, 1.1], the intra-species competition is fixed to ${\beta }_{j}^{P,A}=5$ and the Holling term is ${h}^{P,A}=0.1$. With these parameters, we study the system varying the value of the intensity of the inter-species competition and mutualistic terms, ${\beta }_{0}^{P(A)}$ and ${\gamma }_{0}^{(P)A}$, respectively. For simplicity, we assumed that all the intervening parameters take the same values for plants and animals. Finally, the system is considered to have achieved equilibrium when all the species’ frequencies remain constant. A species is considered to have gone extinct when its relative abundance is lower than ${10}^{-9}$. Each point of Figs 2–4 correspond to 100 simulations with different initial conditions in the abundances and ${\alpha }_{i}^{P,A}$.

### Evolution of the abundance of generalist and specialist species

In order to understand how the population dynamics of generalists and specialists is affected by mutualistic and competitive terms, let us first consider the effect of the mutualist term (the fourth term in equations 1 and 3). This term reads: $\frac{{M}_{i}^{P}}{1+\delta {M}_{i}^{P}}$, where $\delta =h{\gamma }_{0}$. As ${M}_{i}^{P}$ is larger for generalists than for specialists, the increasing rate of the former is stronger than that of the latter. In other words, this term favours the increase of generalists species with respect to specialists ones. The analysis of the inter-species competition term (the third term of equations 1 and 3) is less straightforward. Let us compare the behavior of this term for a generalist and a specialist plant species, of relative abundances ${s}_{1}^{P}$ and ${s}_{2}^{P}$, respectively, in the particular case where the specialist interacts with only one animal species, $l$, of abundance ${s}_{l}^{A}$. This means that ${K}_{2k}={\delta }_{kl}$ and ${M}_{2}^{P}={s}_{l}^{A}$. In this extreme case, it is very easy to see that the competing term that enters in equation 1 for species $2$ reads:

4 $${C}_{2}={\beta }_{0}^{P}\{{s}_{1}^{P}+\sum _{j\in P,j\ne 1,2}{s}_{j}^{P}{K}_{jl}\}$$

where the first term corresponds to its competition with the generalist species $1$, and the second term stands for the competition with all the other plant species that share the same pollinator, $l$, of abundance ${s}_{l}^{A}$. The corresponding competition term for the generalist $1$ is

5 $${C}_{1}={\beta }_{0}^{P}\sum _{j\in P,j\ne 1}{s}_{j}^{P}\{\frac{\sum _{k\in A}{K}_{1k}{K}_{jk}{s}_{k}^{A}}{\sum _{k\in A}{K}_{1k}{s}_{k}^{A}}\}$$

where the factor between braces is ${\alpha }_{j}\le 1$. The latter expression can be rewritten as:

6 $${C}_{1}={\beta }_{0}^{P}({s}_{2}^{P}{\alpha }_{2}+\sum _{j\in P,j\ne 1,2}{s}_{j}^{P}{\alpha }_{j})$$

The second term of equation 6, which may include other generalist plants (those that grow faster with ${\gamma }_{0}$), is reduced by the factors ${\alpha }_{j}\le 1$, and in general ${C}_{2} > {C}_{1}$. Therefore, we can then expect that this unbalance in the corresponding competition terms becomes a supplementary advantage for the generalists, thus reinforcing their growth rate. A similar analysis to estimate the relative importance of the competition term can be done using the properties of projected matrices (see section 5 of Supplementary Information)^49^.

## Electronic supplementary material

Supplementary Information
