# Supplementary material for: The joint influence of competition and mutualism on the biodiversity of mutualistic ecosystems
Source: Sci Rep. 2018 Jun 18;8:9253. doi: 10.1038/s41598-018-27498-8 (PMC6006315; doi:10.1038/s41598-018-27498-8)
Supplement: Supplementary file 1 — Supplementary Information [file 41598_2018_27498_MOESM1_ESM.pdf]

Supplementary information.

The joint influence of competition and mutualism on the biodiversity of mutualistic ecosystems.

Carlos Gracia-Lázaro[1], Laura Hernández[2], Javier Borge-Holthoefer[3], and Yamir Moreno[1,4,5]

[1]Institute for Biocomputation and Physics of Complex Systems (BIFI), University of Zaragoza, Spain
[2]Laboratoire de Physique Théorique et Modélisation, UMR CNRS, Université de Cergy-Pontoise, 2 Avenue Adolphe Chauvin, F-95302, Cergy-Pontoise Cedex, France
[3]Internet Interdisciplinary Institute (IN3), Universitat Oberta de Catalunya, Spain
[4]Department of Theoretical Physics, Faculty of Sciences, University of Zaragoza, Spain
[5]Complex Networks and Systems Lagrange Lab, Institute for Scientific Interchange, Turin, Italy

March 6, 2018

1 Multilayer Networks

A graph (*i.e.*, a single-layer network) is a tuple $G = (\mathcal{V}, E)$, where $\mathcal{V}$ stands for the set of nodes and $E \subset \mathcal{V} \times \mathcal{V}$ is the set of edges that connect pairs of nodes. The edges of the graph induce a binary relation on $\mathcal{V}$ that is called the adjacency relation of G. This relation can be represented through the adjacency matrix $\mathbf{A}$, where $A_{i,j}$ indicates the number of links from node i to node j.

As a particular case, a bipartite graph is a graph whose nodes can be divided into two disjoint sets V_1 and V_2 ($\mathcal{V} = V_1 \cup V_2$, $V_1 \cap V_2 = \emptyset$) such that every edge connects a node in V_1 to one in V_2. The adjacency matrix of a bipartite graph has the form of a block matrix:

$$\mathbf{A} = \begin{bmatrix} \mathbf{O}_{n\times n} & \tilde{\mathbf{A}}_{n\times m} \\ (\tilde{\mathbf{A}}^T)_{m\times n} & \mathbf{O}_{m\times m} \end{bmatrix} \quad , \tag{1}$$

where $\mathbf{O}$ represents the $n \times n$ null matrix, $O_{ij} = 0$.

A multilayer network is a data structure made of multiple layers, where each layer is a single-layer network. Formally, a multilayer network is a tuple $M = (\mathcal{G}, C)$ where $\mathcal{G} = \{G_\alpha; \alpha \in \{1, ..., n\}\}$ is a family of graphs $G_\alpha = (V_\alpha, E_\alpha)$ (the layers), and $C \in V_\alpha \times V_\beta$ $(\alpha, \beta = 1, 2, \ldots, n; \alpha \neq \beta)$ is the set of edges between nodes of different layers.

In order to build a more complete network containing the interactions among species of different guilds as well as intra-guild interactions, we can further exploit the information contained in the bipartite network of mutualistic interactions and consider the projections of the mutualistic interactions onto the guilds of plants (P) and animals (A). This set-up gives us a multilayer network where the guilds constitute the layers, the mutualistic interactions provide the interlayer links and their projections account for the intra-layer links, which in turn represent the number of pollinators (*resp.*, plants) shared by the corresponding plants (*resp.*, pollinators). Accordingly, the resulting multilayer network consists of two layers (V_P, V_A), the set of inter-layer links ($C \in V_P \times V_A$), and two sets of intra-layer links ($E_P \in V_P \times V_P$, $E_A \in V_A \times V_A$).

For the sake of clarity, here we adopt the notation commonly used in the biological literature. The mutualistic plant-pollinator interactions (*i.e.*, the inter-layer connections) are given by a bipartite $N^P \times N^A$ matrix, $\mathbf{K}$, with $K_{ik} = 1$ if animal species k pollinates the plant species i, and $K_{ik} = 0$ otherwise. The projection of this matrix onto the sets of plants is given by:

$$V_{ij}^P = \sum_{k \in A} K_{ik} K_{jk} \quad , \tag{2}$$

where V_{ij}^P represents the number of pollinators shared by plant species i and j.

In our dynamical equations, we consider the resources that are shared by species of the same guild. According to equation 2, the abundance of the pollinators shared by plant species i, j is given by:

$$W_{ij}^P = \sum_{k \in A} K_{ik} K_{jk} s_k^A \quad . \tag{3}$$

Equivalently, the respective formulas for pollinators are obtained by interchanging the P and A superscripts and vice versa, that is,

$$V_{kl}^A = \sum_{i \in P} K_{ik} K_{il} \quad , \tag{4}$$

$$W_{kl}^A = \sum_{i \in P} K_{ik} K_{il} s_i^P \quad . \tag{5}$$

Tables 1 and 2 show the main features (number of species, plants and animals), the location of the biotope and the reference of the ecological networks used to numerically solve the differential equations. Table 1 refers to plant-pollinator networks and table 2 to seed-dispersal networks. All the matrices have been obtained from the *web-of-life.es* database.

network	species	plants	pollinators	location	reference
M-PL-005	371	96	275	Pikes Peak, Colorado, USA	[1]
M-PL-010	107	31	76	Zackenberg, Greenland	*
M-PL-011	27	13	14	Mauritius Island, Mauritius	[2]
M-PL-016	205	26	179	Doñana Nat. Park, Spain	[3]
M-PL-023	95	23	72	Rio Blanco, Mendoza, Argentina	[4]
M-PL-030	81	28	53	Guarico State, Venezuela	[5]
M-PL-032	40	7	33	Brownfield, Illinois, USA	[6]
M-PL-044	719	110	609	Amami-Ohsima Island, Japan	[7]
M-PL-046	60	16	44	Denmark	[8]
M-PL-048	266	30	236	Denmark	[9]
M-PL-054	431	113	318	Kyoto, Japan	[10]
M-PL-056	456	91	365	Mt. Kushigata, Japan	[11]

Table 1: **Plant-pollinator netwoks.** Values for the number of species, plants and animals, location of the study and reference of the plant-pollinator ecological networks used in this study. * M-PL-010: Elberling and Olesen. This network is available as M-PL-010 on the *web-of-life.es* database.

network	species	plants	seed-dispersers	location	reference
M-SD-019	209	169	40	Monteverde, Costa Rica	[12]
M-SD-022	317	207	110	São Paulo, Brazil	[13]

Table 2: **Seed-dispersal netwoks.** Values for the number of species, plants and animals, location of the study and reference of the seed-dispersal ecological networks used in this study.

2 Role of the heterogeneity in the competition parameters

Here we explore a simplified version of the model given by eq.(1) and (2) of the main text in order to investigate if the obtained results could be just a consequence of heterogeneity in the competition intensity. To do so, we consider a situation where the competition term between species i and j of the same layer reads:$C_{ij} = \beta_{ij}\tilde{V_{ij}}s_j$, $\tilde{V_{ij}}$ is the binary projected matrix corresponding to each guild, as explained in the main text, and β_{ij} are taken from an uniform distribution $(0.05\beta_0, 1.95\beta_0)$. It is worth noticing that this introduces a *quenched disorder*, at a difference with our model where the competition intensity is a dynamical variable.

We show below that introducing heterogeneity in the competition term is not enough to reproduce the results observed in the right panel of Figure 2 of the main text. Figures 1, 2 and 3 represent the species persistence as a function of the inter-species competition and mutualistic parameters (*resp.*, β_0 and γ_0). The rows correspond to different real mutualistic networks, plant-pollinator networks in Fig. 1-2 and seed-dispersal networks in Fig. 3. In all figures, left panels show the results of integrating the dynamical equations when the competitive term is homogeneous $\beta_{ij} = \beta_0$, for all interacting species of a given layer (some of which are also shown in the main text). In the central panels, which correspond to the heterogeneous competition parameter, the same qualitative behavior holds but the final biodiversities are lower than in the homogeneous case. Finally, the right panels correspond to the model defined by equations equations (1) and (2) of the main text, and therefore they represent the case in which the competition terms have been weighted by the shared abundance of pollinators (*resp.*, plants). Again, some panels have been introduced in the main text. A direct comparison of the central and right panels clearly shows that the obtained results are not just a mere consequence of heterogeneity in the competition parameter, but they stem from the dynamical character of the competition term associated to the network structure.

3 Robustness of the model

Real mutualistic networks exhibit a wide range of size, connectance and nestedness. The connectance of a network is defined as the fraction of actual mutualistic links with respect to the total possible links in the network: $N^P \times N^A$. Among the different metrics to measure the nestedness, here we use the NODF metric [15], which is bounded to the $[0, 1]$ interval, where higher values indicate higher nestedness. In order to test the robustness of the proposed dynamics under the election of the network, we chose for this study real networks covering a wide range of size, connectance and nestedness. As shown in tables 1 and 2, the size of the selected networks varies from 27 to 719 species. Figure 4 represents the connectance as a function of the nestedness for all the plant-pollinator networks on the *web-of-life.es* database, highlighting the correlation between connectance and nestedness. The networks studied here are shown by red squares.

We have also checked that the results are not dependent on the value of the Holling constant that controls the saturation of the mutualist term. To do so we have integrated the dynamical equations for different values of h. Figure 5 represents the species persistence as a function of the inter-species competition and mutualistic terms (*resp.*, β_0 and γ_0). The different rows correspond to the different mutualistic networks shown in the main text, while each column corresponds to a different value of the Holling constant $h = 0.1, 0.5, 0.9$. As **it** can be seen, the qualitative behavior remains unchanged under the variation of h, that is, regardless the choice of h, in the high-competition regime an increase in mutualism involves a decrease in persistence.

4 Additional intra-species competition term

The model described by equations (1) and (2) of the main text implies that the competition between two plants (respectively, pollinators) is proportional to the abundance of the shared pollinators (respectively, plants). Nevertheless, there are situations in which competition is not driven exclusively by pollination, as in the case of competition for abiotic resources like water, light or nutrients. In order to take into account this fact, here we explore the addition of an extra inter-species competition term to the equation (1) of the main text. As this extra competition term is not related to the projected networks, each species competes with all the others in the layer, with a uniform parameter β_c.

According to this alternative formulation, the equation that describes the evolution of the relative abundances of a given plant i now takes the form:

$$\frac{1}{s_i^P}\frac{ds_i^P}{dt} = \alpha_i^P - \beta_i^P s_i^P - \beta_c^P \sum_{j\in P, i\neq j} s_j^P - \beta_0^P \frac{\sum_{j\in P, i\neq j} s_j^P W_{ij}^P}{M_i^P} + \gamma_0^P \frac{M_i^P}{1 + h^P \gamma_0^P M_i^P} \ . \tag{6}$$

The rest of the terms remain invariant with respect to equation (1) of the main text: the first term of equation 6 represents the intrinsic growth of species i, the second term refers to the intra-specific competition term (saturation), the fourth term accounts for the inter-specific competition for pollinators and, finally, the fifth term gives the contribution of mutualism.

Figure 6 shows the species persistence as a function of the inter-species competition and mutualistic terms (*resp.*, β_0 and γ_0). The rows correspond to different real mutualistic networks as indicated. Left panels show the results of the approach described in this section, that is, when an extra inter-species competitive term not driven by pollinators is added to the equation for the evolution of plant species. In these simulations, the value of β_c^P has been chosen equal to the one used for the pollinators-driven competition β_0^P. Right panels of Fig. 6 correspond to the model defined by equations (1) and (2) of the main text, according to which the inter-specific competition is driven exclusively by the species of the other guild.

As can be seen by comparing the results of both approaches, the introduction of the additional term does not have a qualitative effect on the system's behavior, what means that the results are robust with respect to this modification. Similar results are obtained when the additional term described in equation 6 is introduced in the evolution of animal species or in both guilds.

5 Approximated analysis of the behaviour of the competition term.

Here we discuss an approximated version of the heuristic argument provided in the Methods section of the main text which provides a simple interpretation of the reasons why the structured competition term affects generalists and specialists in different ways. Let us consider the evolution of the abundance of a plant species i, s_i^P. The factor W_{ij}^P of equation 3 involves the abundance of each animal species of the other guild. These abundances are all different because for the animal guild, generalists and specialists also grow faster and slower with γ_0, respectively. We will then consider an average relative abundance for animals $\langle s^A \rangle$. Then the competing term, for plants, reads:

$$C_i \approx \beta_0^P \sum_{j \in P, j \neq i} s_j^P \{ \frac{\langle s^A \rangle V_{ij}^P}{\sum_{k \in A} K_{ik} \langle s^A \rangle} \} \tag{7}$$

where V_{ij}^P is the projected matrix on the plant space.

As $V_{ij}^P \leq min(k_i^P, k_j^P)$ we can consider two situations:

- i is a generalist so $k_i^P \geq k_j^P \forall j$ then, the maximum possible contribution to the competition term is : $C_{gen}^{MAX} \approx \beta_0^P \sum_{j \in P, j \neq i} (s_j^P \frac{k_j^P}{k_i^P})$
- i is a specialist, so $k_i^P < k_j^P$ then the maximum contribution to the competition term is : $C_{spe}^{MAX} \approx \beta_0^P \sum_{j \in P, j \neq i} s_j^P$

which shows that the specialists are, on average, more affected by competition than generalists when mutualism increases. The same reasoning holds symmetrically for the animal's guild.

References

[1] Clements RE, Long FL (1923) *Experimental pollination: An outline of the ecology of flowers and insects.* (Carnegie Institution of Washington, Washington, DC, USA).

[2] Olesen, J. M., Eskildsen, L. I., & Venkatasamy, S. Invasion of pollination networks on oceanic islands: importance of invader complexes and endemic super generalists. *Diversity and Distributions*, **8**(3), 181-192 (2002).

[3] Herrera, J. Pollination relationships in southern Spanish Mediterranean shrublands. *J. Ecol.* **76,** 274-287 (1988).

[4] Medan, D., Montaldo, N. H., Devoto, M., Mantese, A., Vasellati, V., Roitman, G. G. & Bartoloni, N. H. Plant-pollinator relationships at two altitudes in the Andes of Mendoza, Argentina. *Arctic, Antarctic, and Alpine Research*, **34**(3), 233-241 (2002).

[5] Ramirez, N. & Brito, Y. Pollination biology in a palm swamp community in the Venezuelan Central Plains. *Botanical Journal of the Linnean Society*, **110**(4), 277-302 (1992).

[6] Schemske, D. W., Willson, M. F., Melampy, M. N., Miller, L. J., Verner, L., Schemske, K. M., & Best, L. B. Flowering ecology of some spring woodland herbs. *Ecology*, **59**(2), 351-366 (1978).

[7] Kato, M. Anthophilous insect community and plant-pollinator interactions on Amami Islands in the Ryukyu Archipelago, Japan. *Contributions from the Biological Laboratory, Kyoto University* **29,** 157-254 (2000).

[8] Bundgaard, M. Tidslig og rumlig variation i et plante-bestøvernetværk (Doctoral dissertation, MSc. thesis, Aarhus University, Aarhus. FROM BROADSTONE TO ZACKENBERG 57) (2003).

[9] Dupont, Y.L. & Olesen, J.M. Ecological modules and roles of species in heathland plant-insect flower visitor networks. *J.Anim. Ecol.* **78,** 346-353 (2009).

[10] Kakutani, T., Inoue, T., Kato, M. & Ichihashi, H. Insect-flower relationship in the campus of Kyoto University, Kyoto: an overview of the flowering phenology and the seasonal pattern of insect visits. *Contributions from the Biological Laboratory, Kyoto University* **27**, 465 (1990).

[11] Kato, M. *et al.* Flowering phenology and anthophilous insect community in the cool-temperate subalpine forests and meadows at Mt. Kushigata in the central part of Japan. *Contributions from the Biological Laboratory, Kyoto University* **28,** 119 (1993).

[12] Wheelwright, N.T., Haber, W.A., Murray K.G. & Guindon, C. Tropical fruit-eating birds and their food plants: a survey of a Costa Rican lower montane forest. *Biotropica* **16,** 173-192 (1984).

[13] Silva, W.R., Marco J., P. de, Hasui, E. & Gomes, V.S.M. Patterns of fruit-frugivores interactions in two Atlantic Forest bird communities of South-eastern Brazil: implications for conservation. Pp. 423-435. In: D.J. Levey, W.R. Silva and M. Galetti (eds.) *Seed Dispersal and Frugivory: Ecology, Evolution and Conservation* (CABI, 2002).

[14] Bastolla, U. *et al.* The architecture of mutualistic networks minimizes competition and increases biodiversity. *Nature* **458,** 1018-1020 (2009).

Almeida-Neto M, Guimarães P, Guimarães PR, Loyola RD, Ulrich W (2008) A consistent metric for nestedness analysis in ecological systems: reconciling concept and measurement. *Oikos* 117(8):1227-1239.

[15] Almeida-Neto, M., Guimarães, P., Guimarães, P.R., Loyola, R.D. & Ulrich, W. A consistent metric for nestedness analysis in ecological systems: reconciling concept and measurement. *Oikos* **117**(8), 1227-1239 (2008).

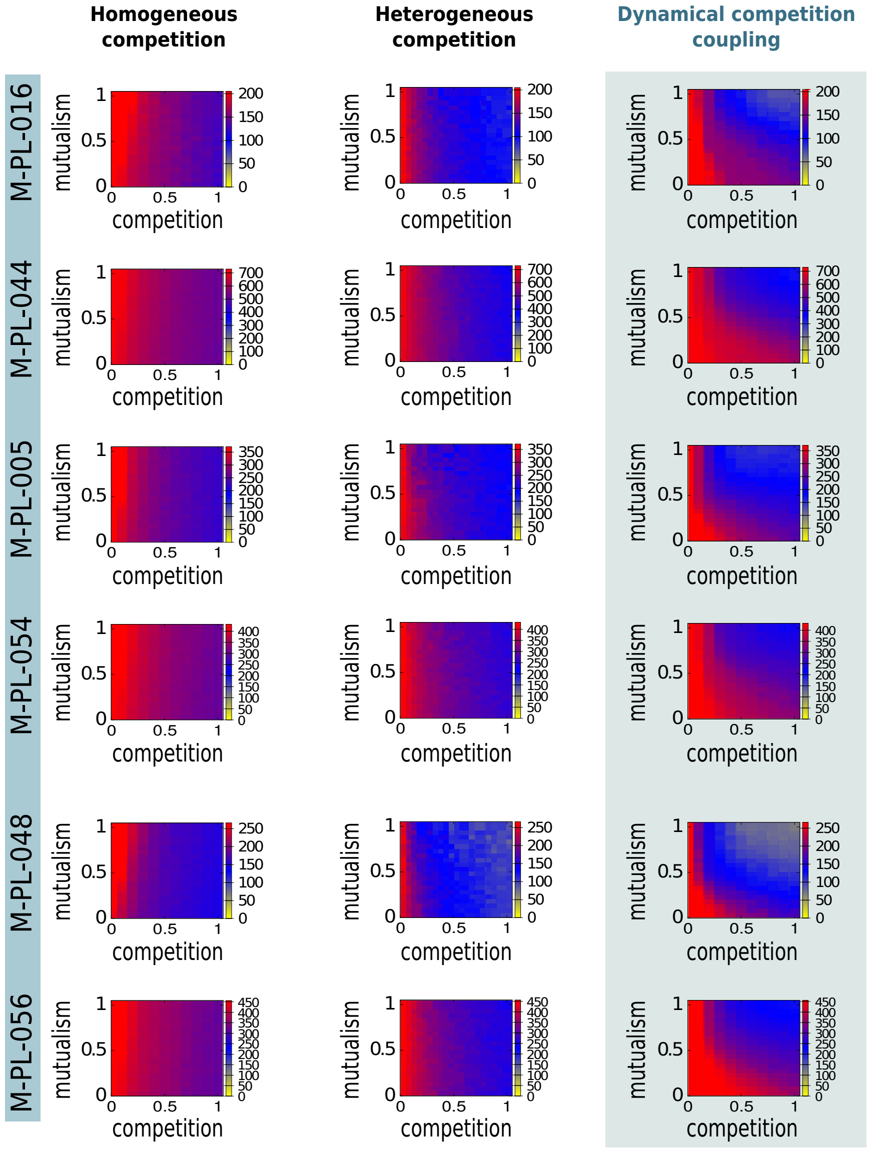

Figure 1: **Biodiversity in plant-pollinator networks under different treatments of the competition term.** The color-coded values in the figure represent the number N of final species as a function of the inter-species competition and mutualistic parameters (*resp.*, β_0 and γ_0), for different mutualistic networks (rows), after the system has evolved according to the dynamical population model. Left panels: results for the homogeneous competition model. All interacting pairs of species in the layer have the same interacting constant $\beta_{ij} = \beta_0 \forall (i,j)$. Central panels: results for the heterogeneous competition model. Interacting constants are drawn from a uniform distribution $\beta_{ij} \in [0.05\beta_0, 1.95\beta_0]$. Finally, the right panels stand for the model given by eq. (1) and (2) of the main text. The maximum diversity, corresponding to no extinctions, is $N = 205, 719, 371, 431, 266, 456$ species for networks M-PL-016, M-PL-044, M-PL-005, M-PL-054, M-PL-048 and M-PL-056 respectively. Other values are: intra-specific competition terms $\beta_i^P = \beta_k^A = 5.0$, growing terms $\alpha_i^P \in (0.9, 1.1)$, $\alpha_k^A \in (0.9, 1.1)$ and Holling terms $h^P = h^A = 0.1$.

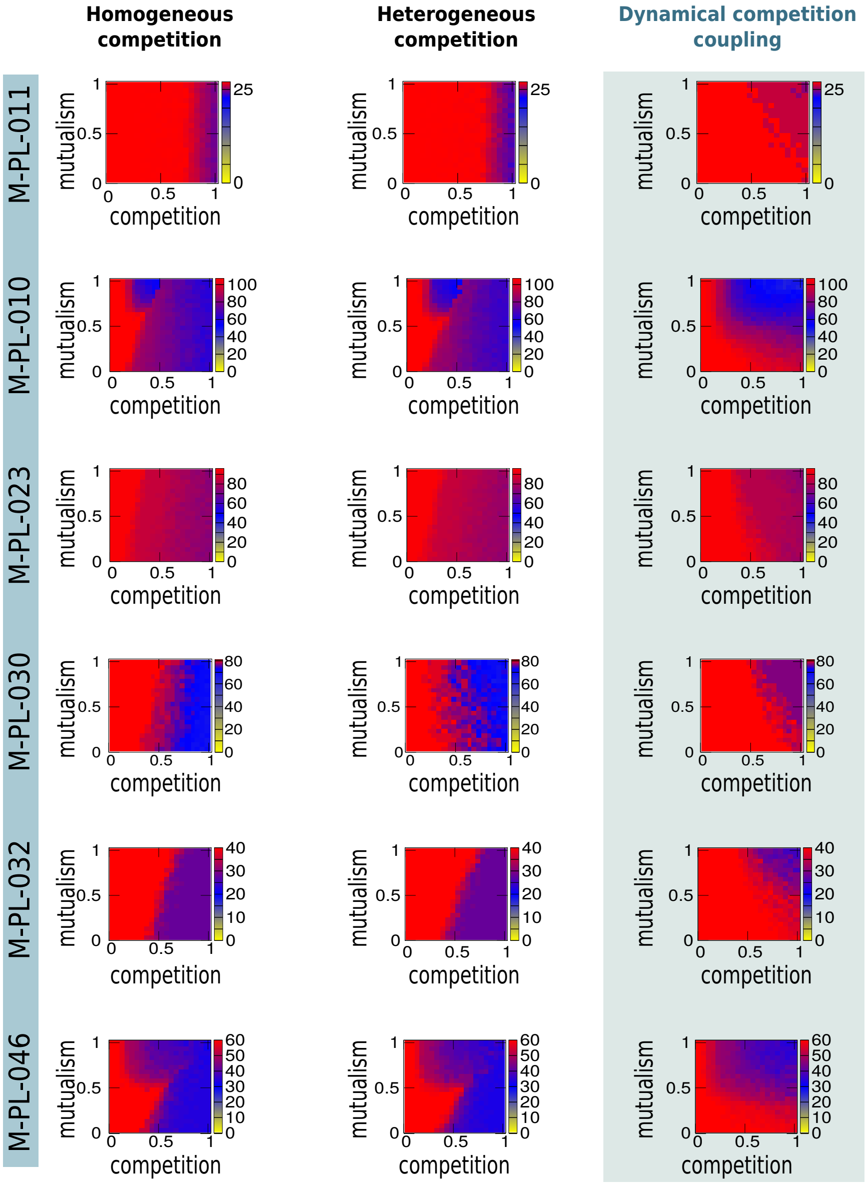

Figure 2: **Biodiversity in plant-pollinator networks under different treatments of the competition term.** Number N of final species as a function of the inter-species competition and mutualistic parameters (*resp.*, β_0 and γ_0), for different mutualistic networks (rows). Left panels: results for the homogeneous competition model. All interacting pairs of species in the layer have the same interacting constant $\beta_{ij} = \beta_0 \forall (i,j)$. Central panels: results for the heterogeneous competition model. Interacting constants are drawn from a uniform distribution $\beta_{ij} \in [0.05\beta_0, 1.95\beta_0]$. Finally, the right panels stand for the model given by eq. (1) and (2) of the main text. The maximum diversity corresponds to $N = 27, 107, 95, 81, 40, 60$ species for networks M-PL-011, M-PL-010, M-PL-023, M-PL-030, M-PL-032 and M-PL-046 respectively. Other values are: $\beta_i^P = \beta_k^A = 5.0$, $\alpha_i^P \in (0.9, 1.1)$, $\alpha_k^A \in (0.9, 1.1)$ and $h^P = h^A = 0.1$.

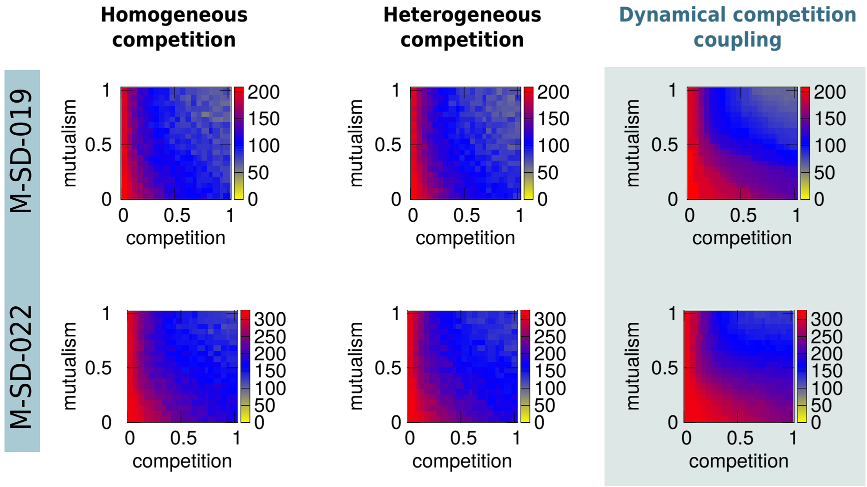

Figure 3: **Biodiversity in seed-dispersal networks under different treatments of the competition term.** Number N of species in the steady state as a function of the inter-species competition and mutualistic parameters (*resp.*, β_0 and γ_0), for different seed-dispersal networks (rows). Left panels: results for the homogeneous competition model. All interacting pairs of species in the layer have the same interacting constant $\beta_{ij} = \beta_0 \forall (i, j)$. Central panels: results for the heterogeneous competition model. Interacting constants are drawn from a uniform distribution $\beta_{ij} \in [0.05\beta_0, 1.95\beta_0]$. Finally, the right panels stand for the model given by eq. (1) and (2) of the main text. The maximum diversity corresponds to $N = 209$ (*resp.*, 318) species for network M-SD-019 (*resp.*, M-SD-022). Other values are: $\beta_i^P = \beta_k^A = 5.0$, $\alpha_i^P \in (0.9, 1.1)$, $\alpha_k^A \in (0.9, 1.1)$ and $h^P = h^A = 0.1$.

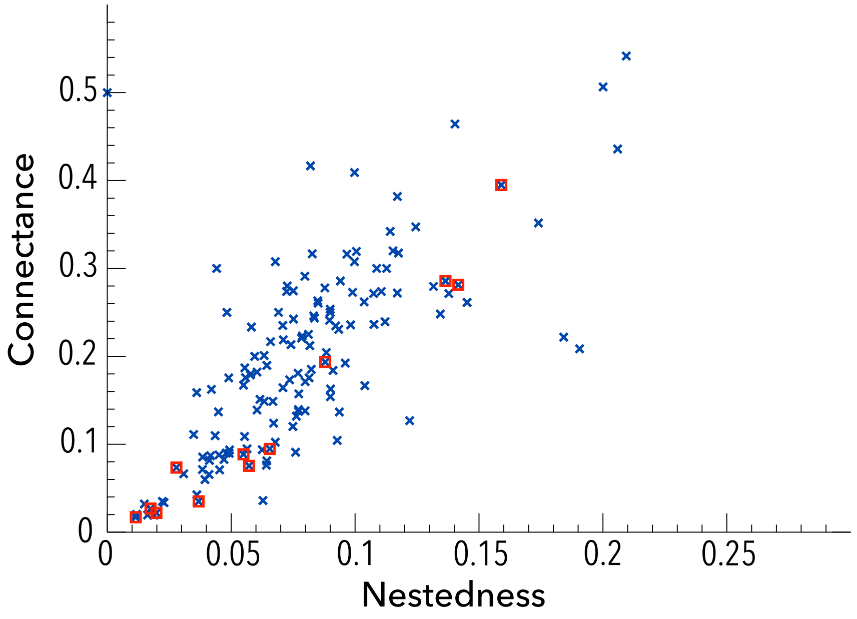

Figure 4: **Connectance and nestedness of mutualistic networks.** Connectance as a function of the nestedness. Blue X's represent the values for all the plant-pollinator networks on the *web-of-life.es* database, while red squares represent the networks selected for this study.

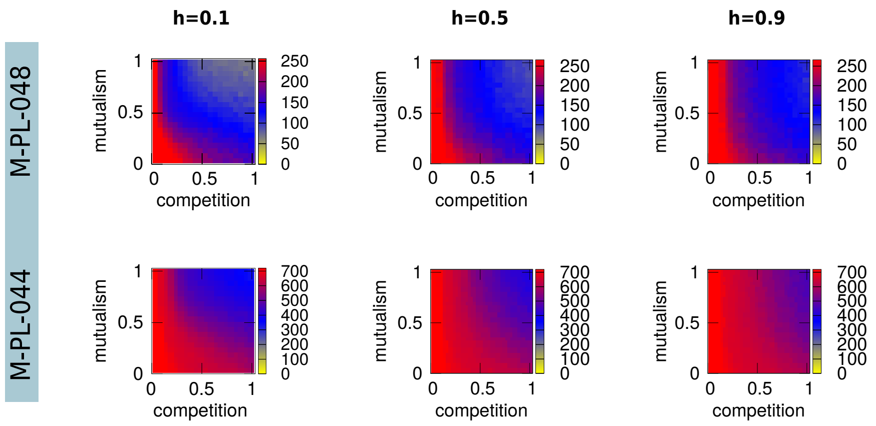

Figure 5: **Biodiversity in plant-pollinator networks for different values of the saturation term.** Number N of final species as a function of the inter-species competition and mutualistic terms (*resp.*, β_0 and γ_0), for different mutualistic networks (rows). Different columns correspond to different values of the Holling term $h = 0.1, 0.5, 0.9$. All the panels correspond to the model described by eqs. (1) and (2) of the main text. The maximum diversity corresponds to $N = 719, 266$ species for networks M-PL-044 and M-PL-048 respectively. Other values are: $\beta_i^P = \beta_k^A = 5.0$, $\alpha_i^P \in (0.9, 1.1)$ and $\alpha_k^A \in (0.9, 1.1)$.

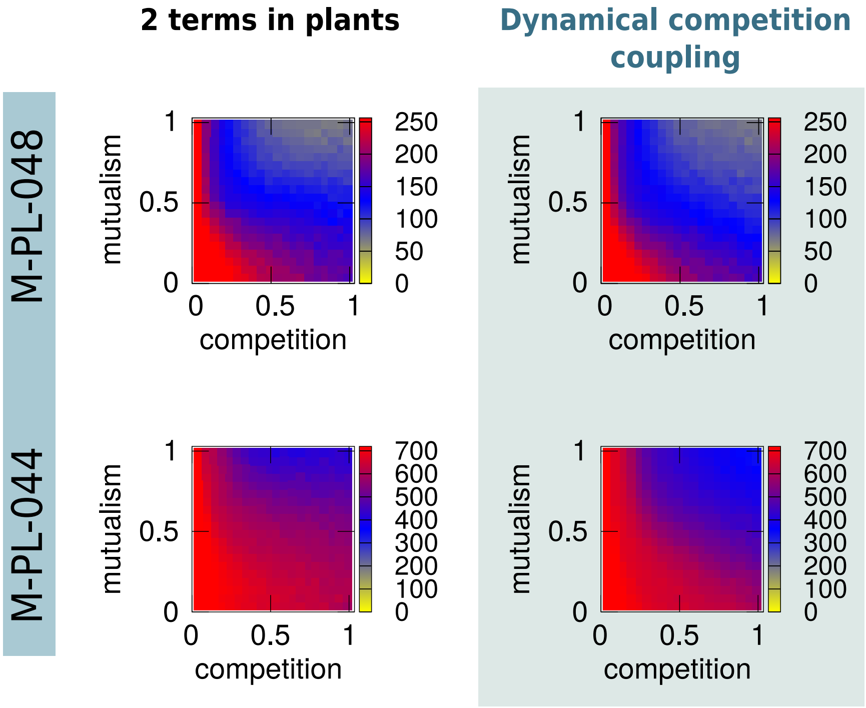

Figure 6: **Additional term in the intra-species competition.** Number N of species in the steady state as a function of the inter-species competition and mutualistic terms (*resp.*, β_0 and γ_0), for different mutualistic networks (rows). Left panels show the results when a mean-field term for the intra-species competition is added to the model given by eqs. (1) and (2) of the main text. Right panels stand for the model here presented. The maximum diversity corresponds to $N = 266$ (*resp.*, 719) species for network M-PL-044 (*resp.*, M-SD-044). Other values are: $\beta_i^P = \beta_k^A = 5.0$, $\alpha_i^P \in (0.9, 1.1)$, $\alpha_k^A \in (0.9, 1.1)$, $\beta_c^P = \beta_0^P$ and $h^P = h^A = 0.1$.
